# Assessments of CYP‑inhibition‑based drug–drug interaction between vonoprazan and poziotinib *in vitro* and *in vivo*

**DOI:** 10.1080/13880209.2023.2173253

**Published:** 2023-02-02

**Authors:** Shan Zhou, Fang-Ling Zhao, Shuang-Hu Wang, Yi-Ran Wang, Yun Hong, Quan Zhou, Pei-Wu Geng, Qing-Feng Luo, Jian-Ping Cai, Da-Peng Dai

**Affiliations:** a The Key Laboratory of Geriatrics, Beijing Institute of Geriatrics, Institute of Geriatric Medicine, Chinese Academy of Medical Sciences, Beijing Hospital, National Center of Gerontology of National Health Commission, Beijing, China; b Peking University Fifth School of Clinical Medicine, Beijing, China; c Laboratory of Clinical Pharmacy, The Sixth Affiliated Hospital of Wenzhou Medical University, The People’s Hospital of Lishui, Lishui, China; d Department of Gastroenterology, Beijing Hospital, National Center of Gerontology, Beijing, China; e Institute of Geriatric Medicine, Chinese Academy of Medical Sciences, Beijing, China

**Keywords:** CYP3A4, pharmacokinetics, drug inhibition, UPLC-MS/MS

## Abstract

**Context:**

Poziotinib and vonoprazan are two drugs mainly metabolized by CYP3A4. However, the drug-drug interaction between them is unknown.

**Objective:**

To study the interaction mechanism and pharmacokinetics of poziotinib on vonoprazan.

**Materials and methods:**

*In vitro* experiments were performed with rat liver microsomes (RLMs) and the contents of vonoprazan and its metabolite were then determined with UPLC-MS/MS after incubation of RLMs with vonoprazan and gradient concentrations of poziotinib. For the *in vivo* experiment, rats in the poziotinib treated group were given 5 mg/kg poziotinib by gavage once daily for 7 days, and the control group was only given 0.5% CMC-Na. On Day 8, tail venous blood was collected at different time points after the gavage administration of 10 mg/kg vonoprazan, and used for the quantification of vonoprazan and its metabolite. DAS and SPSS software were used for the pharmacokinetic and statistical analyses.

**Results:**

*In vitro* experimental data indicated that poziotinib inhibited the metabolism of vonoprazan (IC_50_ = 10.6 μM) in a mixed model of noncompetitive and uncompetitive inhibition. The inhibitory constant K_i_ was 0.574 μM and the binding constant αK_i_ was 2.77 μM. *In vivo* experiments revealed that the AUC_(0-_*_T_*_)_ (15.05 *vs.* 90.95 μg/mL·h) and AUC_(0-∞)_ (15.05 *vs.* 91.99 μg/mL·h) of vonoprazan increased significantly with poziotinib pretreatment. The MRT_(0-∞)_ of vonoprazan increased from 2.29 to 5.51 h, while the CLz/F value decreased from 162.67 to 25.84 L/kg·h after pretreatment with poziotinib.

**Conclusions:**

Poziotinib could significantly inhibit the metabolism of vonoprazan and more care may be taken when co-administered in the clinic.

## Introduction

Acid-related diseases (ARDs) are common diseases worldwide, which mainly include the peptic ulcer disease (PUD) and the gastroesophageal reflux disease (GERD). For a long period, PUD has been one of the major scourges to humanity, and is associated with a high incidence of morbidity and mortality. However, in the past two decades GERD has replaced PUD as the major reason for physician consultation, due to foregut-related symptoms (Sachs et al. 2014). It was considered that PUD only occurred in the presence of gastric acid, leading to the pronouncement ‘no acid, no ulcer’ (Prout 1974). Obtaining a continuous and stable anti-acid effect within 24 h is the key point for the treatment of acid-related diseases (Mori and Suzuki 2019). At present, proton pump inhibitors (PPIs) and potassium competitive acid blockers (P-CABs) are the main drugs for the treatment of gastric acid-related diseases (Oshima and Miwa 2018). Among them, PPIs are prodrugs and need to be rearranged into their active form with the aid of gastric acid to irreversibly inhibit proton pumps and gastric acid secretion (Marabotto et al. 2020). Therefore, the effect of PPIs is relatively time-delayed and has a short half-life (Martinucci et al. 2017). Compared with PPIs, P-CABs have a higher acid inhibition intensity and longer acid inhibition duration, which makes them have wider clinical application prospects.

Vonoprazan fumarate, a potassium ion competitive acid blocker, is a novel acid suppressor (Garnock-Jones 2015). Clinical and animal experiments (Murakami et al. 2016; Kogame et al. 2017) showed that the prototype of vonoprazan can rapidly relieve gastrointestinal symptoms and has the advantages of more rapid onset, longer half-life, and stronger acid inhibition effect relative to those of the traditional PPIs prazole drugs (Shin and Kim 2013; Sugano 2018). In addition, traditional PPIs are mainly metabolized by CYP2C19 (Kogame et al. 2017; Mori and Suzuki 2019), while vonoprazan is not (Sugimoto et al. 2020; Wang Y et al. 2020). Thus, the highly polymorphic *CYP2C19* has little impact on gastric acid inhibition after vonoprazan administration, and the efficacy and effective dose of vonoprazan does not differ significantly among different individuals, especially in the East Asian populations that have a relatively higher proportion of the defective allele CYP2C19*2 or CYP2C19*3 than other races (Hu et al. 2012).

Previous studies reported that vonoprazan can be metabolized by various metabolic enzymes in human hepatocytes, such as CYP3A4, CYP2B6, CYP2D6, and the non-CYP enzyme SULT2A1, producing the main metabolite M-I and other metabolites such as M-II, M-III, M-IV-SUL, *N*-demethylated and *N*-sulfated vonoprazan (Yoneyama et al. 2016; Kogame et al. 2017; Yamasaki et al. 2017; Sugimoto et al. 2020). It has been reported that M-I, mainly metabolized by CYP3A4, is the most important metabolite and drug-drug interactions might occur when vonoprazan is coadministered with other CYP3A4 metabolized drugs (Yamasaki et al. 2017). For example, voriconazole, a broad-spectrum antifungal drug and a well-known inhibitor of CYP3A4 could significantly inhibit the metabolism of vonoprazan if coadministered in the clinic (Shen et al. 2020).

Poziotinib is a novel tyrosine kinase small molecule inhibitor that targets the rare exon 20 insertion mutations in epidermal growth factor receptor (EGFR) and human epidermal growth factor receptor 2 (HER2) (Kim et al. 2013). Previous studies revealed that poziotinib was beneficial for the treatment of acquired mutations (EGFR) caused by clinically localized non-small cell lung (Rosell and Cardona Zorrilla 2021), breast (Kim et al. 2020), and gastric cancer (Kim et al. 2019). In the clinic, many cancer patients need to take antineoplastic drugs for a long time, and some of them have to take acid suppressive drugs at the same time to cure the gastrointestinal side effects of antitumor drugs. Patients with digestive system tumors, such as gastric cancer, are required to take drugs for both antineoplastic therapy and acid suppression. A previous study reported that poziotinib was mainly metabolized to M1 by CYP3A4 and partly metabolized to M2 by CYP2D6 (Ji et al. 2021). Considering that poziotinib and vonoprazan are both newly developed drugs mainly metabolized by CYP3A4 and have a high possibility of coadministration, it is very important to investigate the potential interactions between them to provide a theoretical basis for their coadministration and dose adjustment in the clinic.

## Materials and methods

### Chemicals and reagents

Poziotinib (purity > 98%) and vonoprazan (purity > 98%) were purchased from Sunflower Technology Development Co. (Beijing, China). The vonoprazan metabolite M-I was obtained from Wuxi Medical Technology Co. (Wuxi, China). Diazepam [internal standard (IS); purity > 98%] was purchased from Tianjin Golden York Pharmaceutical Co. (Tianjin China). Methanol and acetonitrile (chromatographic grade) were obtained from Merck GmbH (Darmstadt, Germany). Formic acid (chromatographic grade) was obtained from Sigma-Aldrich (St. Louis, MO, USA). Ultrapure water was obtained from a Milli-Q water purification system (Millipore, Billerica, MA, USA). Rat liver microsomes (RLMs) were prepared in our laboratory. All other chemicals and biologicals were of analytical grade or higher.

### Animals and treatment

Male Sprague-Dawley (SD) rats, weighting 300 ± 20 g, were provided by the Experimental Animal Centre of Wenzhou Medical University and fed under standard conditions at a temperature of 23 ± 2 °C, a relative humidity of 50 ± 10%, and light for 12 h per day. Before the experiment, no other drugs were administered, and the animals were fasted for 12 h. The experimental procedures and protocols conformed to the animal ethics standards and were approved by the ethics committee of Wenzhou Medical University (Animal Ethics Approval Number: wydw2019-650).

### *In vitro* experiments

Rat liver microsomes (RLMs) were prepared according to the previously reported differential centrifugation method (Eriksson 1978; Wang et al. 2018), and the concentration of total protein in the RLM was determined to be 28 mg/mL by the BCA protein quantitative method. Then, a 200 μL incubation system was established, which consisted of 5 μg RLM, 100 mM phosphate buffer (pH 7.4), 10 μM vonoprazan (close to its Km), and a series gradient concentration of poziotinib (100, 50, 10, 5, 1, 0.1, 0.01 μM). The mixture was preincubated at 37 °C for 5 min, and NADPH (1 mM) was then added to start the reaction after vortexing. The mixture was placed at 37 °C in a water bath for 30 min with shaking, and 200 μL acetonitrile was added to terminate the reaction and precipitate the protein. Diazepam-acetonitrile solution was then added accurately to serve as the internal standard. After vortexing, the mixture was centrifuged at 12,000 rpm for 10 min and the supernatant was transferred to the sample bottle for further separation. Vonoprazan and its metabolite M-I were detected by UPLC-MS/MS according to the previously reported method (Shen et al. 2020). The nonlinear regression equation was fitted with GraphPad Prism 9.0 software to calculate the IC_50_ value of poziotinib.

To better understand the inhibitory mechanism of poziotinib on vonoprazan, a series of gradient concentrations of vonoprazan were used in the reaction, which included 1/4-, 1/2-, 1-, and 2-times the K_m_ value. Similarly, concentrations of 0, 1/4-, 1/2-, 1-, and 2-times the IC_50_ of poziotinib were also included in the experiment. The above mentioned UPLC-MS/MS method was used for the quantification of the vonoprazan metabolite M-I. K_i_ and αK_i_ values were calculated, and the inhibitory mechanism was analyzed using a primary Lineweaver-Burk diagram, Dixon diagram, and secondary Lineweaver-Burk diagram by GraphPad Prism 9.0.

### Pharmacokinetic experiments *in vivo*

Twelve healthy male SD rats were randomly divided into two groups. The poziotinib group was given 5 mg/kg poziotinib by gavage every day for 7 days, and rats in the control group only received equal amounts of 0.5% CMC-Na solution in the pre-treatment time. Then animals in the poziotinib group and the control group were fasted for 12 h. On Day 8, the poziotinib group was given poziotinib (5 mg/kg) by gavage, and vonoprazan (10 mg/kg) was given 30 min later, while the control group was only given vonoprazan (10 mg/kg) by gavage. Tail venous blood (300 µL) was collected at 0.083, 0.25, 0.5, 1, 2, 3, 4, 6, 12, and 24 h after the administration of vonoprazan, and then the blood was centrifuged at 4,000 rpm, and 4 °C for 10 min. The serum was collected and stored at −80 °C. After thawing at room temperature, 100 μL of the serum sample was removed and 200 μL of acetonitrile containing diazepam (500 ng/mL) was then added to precipitate proteins. After vortexing, 150 μL of supernatant was removed and centrifuged at 13,000 rpm for 5 min. Then, 2 μL of sample was used for the detection of vonoprazan and its main metabolite M-I by UPLC-MS/MS.

DAS (version 3.2.8; The People’s Hospital of Lishui) was used to fit and calculate the pharmacokinetic parameters of vonoprazan and its metabolite M-I, which include peak time (*T*_max_), maximum plasma concentration (*C*_max_), elimination half-life (*T*_1/2_), area under the drug-time curve (AUC) and clearance rate (*CLz*/*F*). SPSS (version 25.0, IBM, USA) was used for the data processing. Student’s *t*-test was used to compare the normal distribution of the data, and the Mann–Whitney *U* test was used for the nonnormal distribution of the data. *p < 0.05 indicates a significant difference compared to the control group and **p < 0.001 indicates an extremely significant difference compared to the control group.

### Liquid chromatography and mass spectrometry conditions of vonoprazan, metabolite M-I, and internal standard

ACQUITY I-Class UPLC and Waters XEVO TQD MS (Milford, MA, USA) were used for the detection of vonoprazan and M-I with an ACQUITY UPLC BEH C18 chromatographic column (50 × 2.1 mm, 1.7 μm) at 40 °C. The mobile phase consisted of acetonitrile and 0.1% formic acid in water in gradient proportions, as shown in Table 1, with a flow rate of 0.4 mL/min. The injection volume of the sample was 2 μL. The scanning method was multiple reaction monitoring (MRM) with detection in positive ion mode and an ESI + ion source. Other mass spectrometry parameters were as follows: capillary voltage 2.0 kV, ion source temperature 150 °C, dissolvent temperature 500 °C, argon flow rate 0.15 mL/min, cone hole gas flow 50 L/h, solvent gas flow 1000 L/h. The precursor ion and product ion were *m/z* 346.04→314.97 for vonoprazan, *m/z* 347.50→205.00 for M-I, and *m/z* 285.10→193.10 for internal standard (IS), diazepam.

**Table 1.** Mobile phase ratio.

Time (min)	A% (acetonitrile)	B% (0.1% folic acid)
0.0	10	90
0.6	50	50
1.0	80	20
2.0	95	5
2.5	95	5
2.6	10	90
3.0	10	90

## Results

### *In vitro* effect of poziotinib on vonoprazan

**Figure 1.**
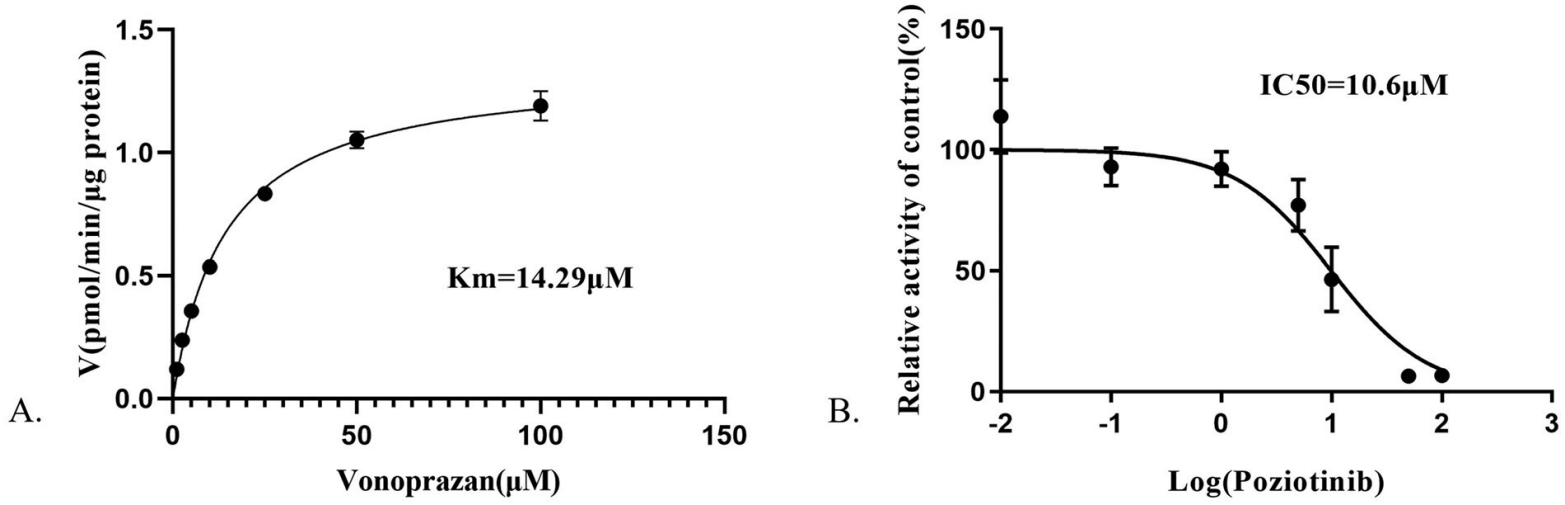
Michaelis–Menten kinetics (A) and the IC50 value (B) of vonoprazan in rat liver microsomes.

**Figure 2.**
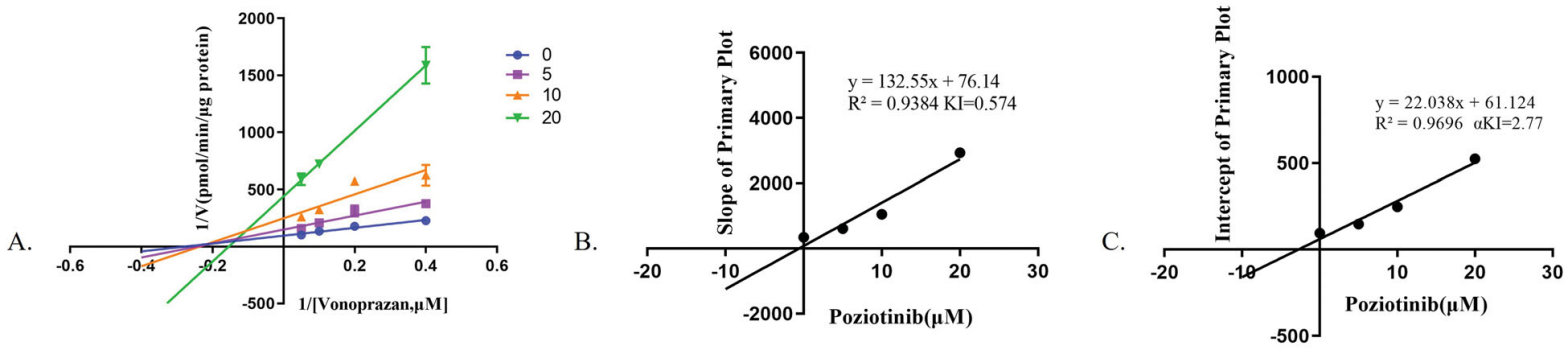
The inhibitory mechanism of poziotinib on vonoprazan. Data shown are the mean ± standard deviation of triplicate experiments. (A) Lineweaver–Burk plots for poziotinib (0, 2.5, 5, 10, 20 μM) inhibition of vonoprazan (0, 5, 10, 20 μΜ) in rat liver microsomes. (B) Slope of the primary plot. (C) Intercept of primary plot.

As shown in Figure 1, the IC_50_ value of poziotinib was 10.6 μM, indicating that poziotinib had an inhibitory effect on vonoprazan *in vitro*. The Lineweaver-Burk plot for poziotinib inhibition of vonoprazan metabolism in rat liver microsomes indicated that this inhibition effect belongs to a mixed uncompetitive and noncompetitive inhibition with K_i_ = 0.574 μM and αK_i_ = 2.77 μM (Figure 2).

### *In vivo* pharmacokinetics detection

**Figure 3.**
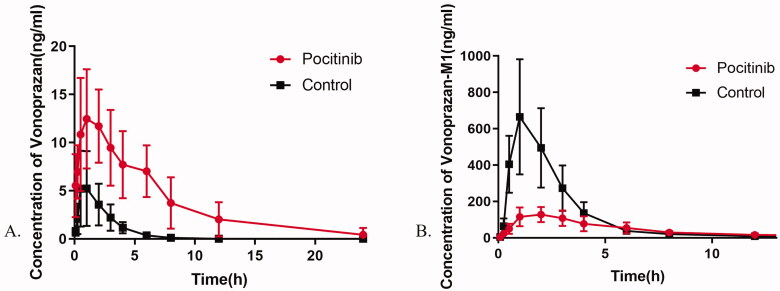
Mean plasma concentration–time profiles of vonoprazan (A) and vonoprazan M-I (B) after an oral administration of vonoprazan (10 mg/kg) to rats in the presence and absence of poziotinib (5 mg/kg). *n* = 6 per group. Data are expressed as mean ± *SD*. The poziotinib group was pretreated with poziotinib (5 mg/kg, oral) before vonoprazan (10 mg/kg, oral) and the control group was administered vonoprazan (10 mg/kg, oral).

The mean plasma concentration–time profiles of vonoprazan and M-I in rats are illustrated in Figure 3, and detailed pharmacokinetic parameters are listed in Tables 2 and 3. Pretreatment with poziotinib led to a significant increase in the values of AUC_(0-_*_T_*_)_, AUC_(0-∞)_, MRT_(0-_*_T_*_)_, MRT_(0-∞)_, and *C*_max_ of vonoprazan, whereas the values of *Vz*/*F* and *CLz*/*F* were significantly decreased compared to those of the control group.

**Table 2.** Mean pharmacokinetic parameters of vonoprazan after oral administration (10 mg/kg) to rats in the presence and absence of poziotinib (5 mg/kg).

Parameters	Control group	Poziotinib group
AUC_(0-_*_t_*_)_ (µg·L^−1^·h^−1^)	15.05 ± 9.22	90.59 ± 41.94**
AUC_(0-∞)_ (µg·L^−1^·h^−1^)	15.05 ± 9.22	91.99 ± 43.84**
MRT_(0-_*_t_*_)_ (h)	2.29 ± 0.36	5.26 ± 1.77**
MRT_(0-∞)_ (h)	2.29 ± 0.36	5.51 ± 2.06*
*t*_1/2_ (h)	1.25 ± 0.77	2.84 ± 1.44
*T*_max_ (h)	0.63 ± 0.31	1.92 ± 2.06
*Vz*/*F* (L·kg ^−1^)	298.47 ± 191.15	90.51 ± 27.33*
*CLz*/*F* (L·h^−1^·kg^−1^)	162.67 ± 63.14	25.84 ± 11.28**
*C*_max_ (µg·L^−1^)	5.69 ± 3.73	15.37 ± 4.98**

AUC: area under the plasma concentration–time curve; CL: plasma clearance; *C*_max_: maximum plasma concentration; MRT: mean residence time; *t*_1/2_: half-life; *T*_max_: time taken to reach maximum plasma level.

*Notes. n* = 6 per group; data are expressed as mean ± *SD*.

*p < 0.05, indicates a significant difference to the control group.

**p < 0.01, indicates an extremely significant difference to the control group.

**Table 3.** Mean pharmacokinetic parameters of M-I after oral administration of vonoprazan (10 mg/kg) to rats in the presence and absence of poziotinib (5 mg/kg).

Parameters	Control group	Poziotinib group
AUC_(0-_*_t_*_)_ (µg·L^−1^·h^−1^)	1319.92 ± 389.57	822.67 ± 348.30*
AUC_(0-∞)_ (µg·L^−1^·h^−1^)	1323.65 ± 388.98	887.11 ± 484.72
MRT_(0-_*_t_*_)_ (h)	2.73 ± 0.39	5.47 ± 1.77*
MRT_(0-∞)_ (h)	2.81 ± 0.39	6.69 ± 4.19
*t*_1/2_ (h)	3.67 ± 0.74	4.31 ± 3.91
*T*_max_ (h)	1.17 ± 0.41	2 ± 1.10
*Vz*/*F* (L·kg ^−1^)	8.76 ± 3.34	15.09 ± 12.80
*CLz*/*F* (L·h^−1^·kg^−1^)	1.64 ± 0.54	2.74 ± 1.20
*C*_max_ (µg·L^−1^)	481.26 ± 196.17	140.75 ± 42.87**

AUC: area under the plasma concentration–time curve; CL: plasma clearance; *C*_max_: maximum plasma concentration; MRT: mean residence time; *t*_1/2_: half-life; *T*_max_: time taken to reach maximum plasma level.

*Notes. n* = 6 per group; data are expressed as mean ± *SD*.

*p < 0.05, indicates a significant difference to the control group.

**p < 0.01, indicates an extremely significant difference to the control group.

Compared with the control group, the AUC_(0-_*_T_*_)_ and AUC_(0-∞)_ of vonoprazan in the poziotinib group were increased to 6.02- and 6.11-fold, with MRT_(0-_*_T_*_)_ and MRT_(0-∞)_ increased to 2.30- and 2.41-fold, respectively. When pre-administered with poziotinib for 7 days, the *C*_max_, *T*_max_, and *T*_1/2_ values of vonoprazan were significantly increased to 2.7-, 3.0-, and 2.2-fold, while the values of *Vz*/*F* and CLz/F were reduced by 70 and 84%, respectively. In contrast, most pharmacokinetic parameters of the main metabolite of vonoprazan, M-I, were reduced when rats were pretreated with poziotinib. Comparing the poziotinib group with the control group, the AUC_(0-_*_T_*_)_, AUC_(0-∞)_, and *C*_max_ values of M-I were reduced by 38, 33, and 71%, respectively. These results demonstrated that pretreatment with poziotinib could inhibit the metabolism of vonoprazan in rats.

## Discussion

Drug-drug interaction (DDI) refers to the simultaneous or sequential administration of more than two drugs in which one drug alters the pharmacological effects of the other (Lee et al. 2015; Liu et al. 2017). When DDI occurs among drugs, administration of a regular dose may increase the drug toxicity or weaken the therapeutic effect of some drugs (Wang J et al. 2020), resulting in the occurrence of adverse drug reactions or treatment failure. Most clinically used drugs are metabolized by cytochrome P450 enzymes in the liver and the intestinal tract, and the inhibition or induction of P450 enzymatic activity is the main cause of metabolic DDIs in the clinic (Benet et al. 2019). According to the recommendation of the FDA, DDI studies should give more attention to seven major CYP isoenzymes: CYP1A2, CYP2B6, CYP2C8, CYP2C9, CYP2C19, CYP2D6 and CYP3A4 (Kato 2020; Wang J et al. 2020). Among them, CYP3A4 is one of the most important isozyme subfamilies, accounting for 40% of all CYP content and metabolizing ∼30–40% of clinically used drugs (Werk and Cascorbi 2014). It also has many active sites that can match substrates with different sizes and chemical properties (Tyzack and Kirchmair 2019; Kato 2020). Specifically, CYP3A4 can bind and oxidize some substrates in a synergistic way, leading to the induction or inactivation of its catalytic activity by these substrates or drugs (Sevrioukova and Poulos 2015). For example, macrolide antibiotics are reported to be a strong inhibitor of CYP3A4 and can cause serious adverse reactions when combined with calcium blockers (Gandhi et al. 2013).

As a new acid suppressor mainly metabolized by CYP3A4, vonoprazan has the advantages of strong acid suppressive ability and lasting effect compared with the traditional acid suppressor PPI (Echizen 2016; Yamasaki et al. 2017). More importantly, PPI is mainly metabolized by the CYP2C19 enzyme and some allelic mutations in the *CYP2C19* gene can affect the metabolic activity of the enzyme, which may lead to different treatment outcomes when using the same drug dose for different individuals (El Rouby et al. 2018). In contrast, the drug curative effect of vonoprazan is not affected by the polymorphic variability of *CYP2C19* (Shin et al. 2011), whereas more attention may be given to the potential drug-drug interactions when combined with other drugs. Suzuki et al. (2003) and Nishihara et al. (2019) proved that clarithromycin, metabolized by CYP3A4 and as a CYP3A4/5 inhibitor, can inhibit the metabolite of vonoprazan. Suzuki et al. (2021) showed that the blood concentration of tacrolimus could significantly increase by 65.8% after switching from rabeprazole, a conventional PPI drug, to vonoprazan coadministration in kidney transplant recipients. These examples remind us that when vonoprazan is used in combination with other drugs, especially for drugs that are also metabolized by CYP3A4, it is necessary to guard against the occurrence of adverse drug reactions, and the dose should be adjusted, or drugs should be changed if necessary.

As a new third-generation tyrosine kinase small molecule inhibitor, poziotinib belongs to the quinazoline derivatives that also include afatinib, erlotinib, gefitinib, dacomitinib, etc. (Kim et al. 2013). It has been reported that quinazoline drugs are mainly metabolized by CYP2D6 and CYP3A4 but exhibit different effects on the metabolic activities of CYP enzymes (Wang J et al. 2020). Dungo and Keating (2013) reported that afatinib had no impact on the catalytic activity of any CYP enzyme. Ma et al. (2015) indicated that erlotinib could inhibit the activities of CYP2B1 and CYP3A1/2 in rats. Prommer (2004) and Hiraide et al. (2019) reported that gefitinib exhibited inhibitory effects on both CYP2C9 and CYP2D6 enzymes. Shirley (2018) showed that dacomitinib was an effective inhibitor of CY2D6. Recently, Wang J et al. (2020) reported that poziotinib could inhibit the activities of CYP2B1 and CYP2C11 in rats but induce the activities of CYP1A2 and CYP2E1. However, no reports have indicated the impact of poziotinib on the activity of CYP3A4 to date.

Considering that both vonoprazan and poziotinib can be metabolized by CYP3A4, some potential drug-drug interactions might occur between them. In this study, the *in vitro* data truly indicated that poziotinib could significantly inhibit the metabolism of vonoprazan through a mixed inhibitory mechanism. In addition, the pharmacokinetic parameters of vonoprazan in poziotinib pretreated rats were significantly increased compared with those in the control group. The serum concentration and peak time of metabolite M-I in the experimental group were much lower than those in the control group, indicating that the metabolite production of vonoprazan in rats was significantly reduced. These data revealed that poziotinib could significantly inhibit the metabolism of vonoprazan both *in vitro* and *in vivo*.

Although our data confirmed the DDIs between vonoprazan and poziotinib, some limitations still exist in this study. First, the poziotinib was regarded as the inhibitor, and vonoprazan was used as the substrate in all the experiments. We are not sure whether vonoprazan also has an inhibitory effect on the metabolism of poziotinib if we set poziotinib as the substrate. Second, we only focused on the quantification of the main metabolite of vonoprazan M-I in this experiment. Yamasaki et al. (2017) pointed out that vonoprazan could be metabolized in two different pathways: one is the oxidative pathway *via* CYP450 such as CYP3A4, CYP2B6, CYP2C19, and CYP2D6, and the other is the nonoxidative pathway *via* SULT2A1. It is believed that CYP3A4 mainly contributed only to the metabolism of vonoprazan to M-I, M-III, and *N*-demethylated TAK-438. Thus, the measurement of other metabolites may be needed for a comprehensive evaluation of the impact of poziotinib on vonoprazan metabolism. Finally, all the experiments were performed in rats, and it has been reported that the metabolites of vonoprazan in rats and humans are not perfectly consistent. Thus, further investigations on humans must be performed to better understand the interactions of poziotinib and vonoprazan in the clinic.

## Conclusions

Our data revealed that poziotinib could significantly inhibit the metabolism of vonoprazan and alter its pharmacokinetic parameters both *in vivo* and *in vitro*. These data indicated that more care may be taken when poziotinib and vonoprazan are coadministered in the clinic, although further clinical investigation is still needed to clarify these potential drug-drug interactions in humans.
